# Symbiotic ß-Proteobacteria beyond Legumes: *Burkholderia* in *Rubiaceae*

**DOI:** 10.1371/journal.pone.0055260

**Published:** 2013-01-25

**Authors:** Brecht Verstraete, Steven Janssens, Erik Smets, Steven Dessein

**Affiliations:** 1 Plant Conservation and Population Biology, KU Leuven, Leuven, Belgium; 2 Naturalis Biodiversity Center, Leiden University, Leiden, The Netherlands; 3 National Botanic Garden of Belgium, Meise, Belgium; University of Vienna, Austria

## Abstract

Symbiotic ß-proteobacteria not only occur in root nodules of legumes but are also found in leaves of certain *Rubiaceae*. The discovery of bacteria in plants formerly not implicated in endosymbiosis suggests a wider occurrence of plant-microbe interactions. Several ß-proteobacteria of the genus *Burkholderia* are detected in close association with tropical plants. This interaction has occurred three times independently, which suggest a recent and open plant-bacteria association. The presence or absence of *Burkholderia* endophytes is consistent on genus level and therefore implies a predictive value for the discovery of bacteria. Only a single *Burkholderia* species is found in association with a given plant species. However, the endophyte species are promiscuous and can be found in association with several plant species. Most of the endophytes are part of the plant-associated beneficial and environmental group, but others are closely related to *B. glathei*. This soil bacteria, together with related nodulating and non-nodulating endophytes, is therefore transferred to a newly defined and larger PBE group within the genus *Burkholderia*.

## Introduction

Almost every plant species on earth interacts in one way or another with endophytic microorganisms [1], and a large amount of literature has been published on bacteria and fungi that colonize internal tissues of plants. In contrast to phytopathogens, endophytic bacteria do not always show external signs of infection, nor do they have a detrimental effect on their host [2]. The interaction between plants and endophytic bacteria in the formation of specialized root nodules is well known and intensively studied for the family *Fabaceae* [3]. Most of the described nitrogen-fixing rhizobia belong to the α-subclass of proteobacteria, but several ß-proteobacterial species of the genus *Burkholderia* are also known to nodulate legumes [4]. Another type of endosymbiosis is found in the family *Rubiaceae* where leaf galls with bacterial endophytes occur in the genera *Pavetta*, *Psychotria* and *Sericanthe* [5]. The presence of endophytic bacteria is visible by eye because of the formation of dark spots or galls in the leaf blades. The endophytes of *Rubiaceae* plants have only been identified recently and all of them belong to *Burkholderia*, a genus that also contains species found in root nodules of legumes [6]–[9]. It has been speculated that *Burkholderia* endophytes are involved in nitrogen fixation and in the production of plant growth regulators, but the recent study of the genome of endophytic *B. kirkii* found no genetic evidence for diazotrophy or for hormone production [10]. Previous phylogenetic analysis of the bacteria showed that each nodulating plant species is colonized by a single *Burkholderia* endophyte [5]. Non-nodulating bacteria that occur free between the mesophyll cells have been found in a few *Fadogia* and *Vangueria* species known to cause a fatal disease in ruminants, called gousiekte [9]. These endophytes differ from the nodulating bacteria by not forming distinct visible galls in the plant leaves. Using a cultivation-independent approach the endophytes were identified as *Burkholderia*, the same genus of bacteria that is found to nodulate other rubiaceous plants [9]. The presence of non-nodulating bacteria in plants formerly not involved in leaf endosymbiosis suggests a wider occurrence of bacterial endophytes. *Fadogia* and *Vangueria* both belong to the tribe *Vanguerieae* (*Rubiaceae*), which makes it an ideal group to screen for the presence of endophytes and to investigate the plant-bacteria association. By doing so, we will document the *Burkholderia* diversity associated with *Rubiaceae* host plants and learn more about the nature of the interaction.

## Materials and Methods

In total 162 specimens of 86 plant species of the tribe *Vanguerieae* (*Rubiaceae*) and 3 outgroup species were gathered from different countries on the African continent (Table S1). All necessary permits for the collection of these plants were obtained and are deposited at the National Botanic Garden of Belgium. The plant data are adapted from the most recent phylogenetic study of *Vanguerieae* and detailed information on their DNA sequences can be found in Verstraete et al. [11].

Host plant leaves were picked in the field and immediately put on silica to allow rapid dehydration and DNA preservation. The leaves, together with the silica, were kept in airtight plastic bags. The silica-dried leaves were handled with sterile tweezers on a sterilized workbench and rinsed with 70% ethanol to remove debris and epiphytes from the leaf surfaces. This technique has been applied successfully and proven to be adequate in previous studies on *Burkholderia* endophytes in Rubiaceae [5]–[9].

To visually demonstrate the presence of bacteria in the leaf blade – and making sure we are dealing with true endophytes – preserved leaves of the host plant *Fadogia homblei* (voucher Lemaire & Verstraete 22, BR) that were collected on 70% ethanol, were investigated with scanning electron microscopy. The leaves were rinsed with fresh 70% ethanol and dissected using razor blades under a stereomicroscope (Wild M3, Wild Heerburgg Ltd). The samples were dehydrated in a 1∶1 mixture of ethanol and dimethoxymethane (DMM), followed by 20 min in 100% DMM. After critical point drying (CPD030, BAL-TEC AG), the dried material was mounted on aluminium stubs using double-adhesive tape and coated with gold (SPI Module Sputter Coater, SPI Supplies). Microscopic observations were made using a JEOL JSM-6360 SEM. To check whether the surface sterilization of the silica-dried leaf samples was successful and we did not sequence epiphytic bacteria, we rinsed a silica-dried leaf sample of *Fadogia homblei* (voucher Lemaire & Verstraete 22, BR) with 70% ethanol, mounted it on a stub, coated it with gold and observed it in the SEM. The dehydration steps of the critical point drying were omitted, because this could additionally remove possible epiphytes and therefore yield false negative results.

Extraction of DNA from the silica-dried leaves of the host plants was performed using the E.Z.N.A.^TM^ HP Plant DNA Mini Kit (Omega Bio-Tek) according to the manufacturer's instructions. Initially, PCR amplification of bacterial 16S rRNA coding gene was preformed using the universal primers 16SB/16SE [12]. A second *Burkholderia* specific reverse primer 16S2, corresponding to position 1262–1285 relative to *Escherichia coli* 16S rRNA gene, was subsequently used to avoid amplification of chloroplast homologues [8]. Amplification primers for *gyrB* and *recA* genes and their respective temperature profiles are based on the protocol in Verstraete et al. [9]. The plant DNA markers are *trnTL*, *trnLF*, *rpl16*, *petD*, *rpl32-trnL*, *accD-psaI* and the ITS region. Further information on the amplification and sequencing of these markers can be found in Verstraete et al. [11].

The sequences were assembled and edited using Geneious 5.4 [13]. All new DNA data are deposited in GenBank and the accession numbers can be found in Table S2. Related sequences of *Burkholderia* spp. were obtained from the BCCM/LMG Bacteria Collection (http://bccm.belspo.be) and GenBank (www.ncbi.nlm.nih.gov/genbank) (Table S2). A preliminary sequence alignment was performed in Geneious followed by manual adjustments resulting in an unequivocal alignment.

Phylogenetic trees were estimated using Bayesian probabilistic methods implemented in MrBayes 3.1 [14], running four Markov chains sampling every 100 generations for five million generations. Preforming jModelTest 0.1.1 [15] resulted in the selection of the following DNA substitution models under the Akaike Information Criterion: GTR+I+G for the 16S rRNA gene and GTR+G for the *gyrB* and *recA* genes. The concatenated dataset was partitioned and independent models were applied for each of the partitions. Maximum parsimony analyses were conducted using Paup* v.4.0b10a [16]. Heuristic searches were conducted with TBR branch swapping on 10 000 random addition replicates with five trees held at each step. Non-parametric bootstrap analysis was carried out to calculate the relative support for individual clades found in the parsimony analysis. For each of 1 000 bootstrap replicates, a heuristic search was conducted with identical settings as in the original heuristic analysis.

Optimization of the presence and absence of the endophytic bacteria on the phylogenetic tree of the host plants was used to investigate the pattern of host-endophyte interaction. Bayesian posterior character mapping was conducted with SIMMAP v1.0 [17] using the obtained Bayesian topologies without burn-in as input data. The approach of Couvreur et al. [18] was used to calculate the hyperparameters that define the mean (E) and standard deviation (SD) that accommodate the substitution rate parameter θ. A flat prior was used for the bias rate parameter I in all analyses. E (θ) and SD (θ) values were independently selected using the “number of realizations sampled from priors” function as implemented in SIMMAP [18]. The mean E (θ) value was optimized at 2 for the presence/absence of an endosymbiotic interaction, whereas the SD (θ) was fixed at 2.

## Results

A molecular, cultivation-independent approach is a useful technique for the detection of bacterial endophytes in plant leaves. In this study, 31 plant species out of the 89 investigated were found to harbour endophytic bacteria inside their leaves (Table S1). Endophytic colonization was not obvious for any of the 76 investigated specimens of these 31 plant species, as they show no external sign of infection.

**Figure 1:**
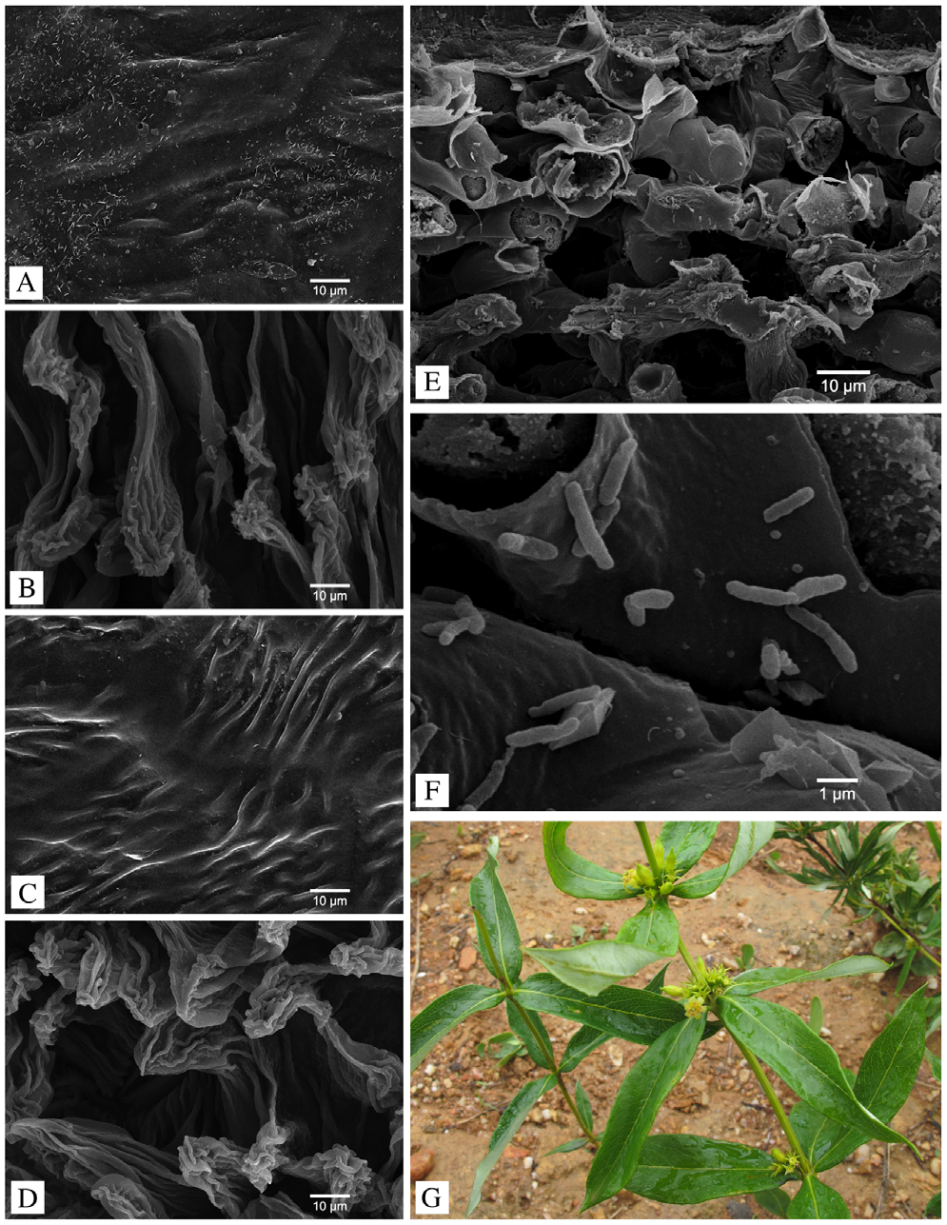
SEM micrographs of the host plant *Fadogia homblei*. A. The upper leaf surface before sterilization with a lot of undefined debris. B. The lower leaf surface before sterilization with some debris present. C. After sterilization the upper leaf surface is almost entirely devoid of particles and no epiphytes are visible. D. On the lower leaf surface there are no epiphytes visible after sterilization. E. Cross section through a leaf showing the mesophyll cells with scattered bacterial endophytes. F. Detail of the endophytes in the intercellular space. G. The host plant *Fadogia homblei* does not have dark bacterial galls on its leaf blades.

To show the endophytic lifestyle of this group, we used scanning electron microscopy to visualize the bacteria inside the leaf blades of the host plant *Fadogia homblei* (Fig. 1). Before surface sterilization a lot of debris is present on both upper and lower leaf surface (Fig. 1A, B). However, no epiphytic bacteria were found after surface sterilization (Fig. 1C, D). Should some external contamination still have occurred, the excessive number of endophytes compared to a single epiphytic bacterium ensures that the DNA results can be interpreted unambiguously. The endophytes are scattered between the mesophyll cells (Fig. 1E, F) and are not housed in specialized leaf galls (Fig. 1G), as sometimes seen in other *Rubiaceae* host plants.

**Figure 2:**
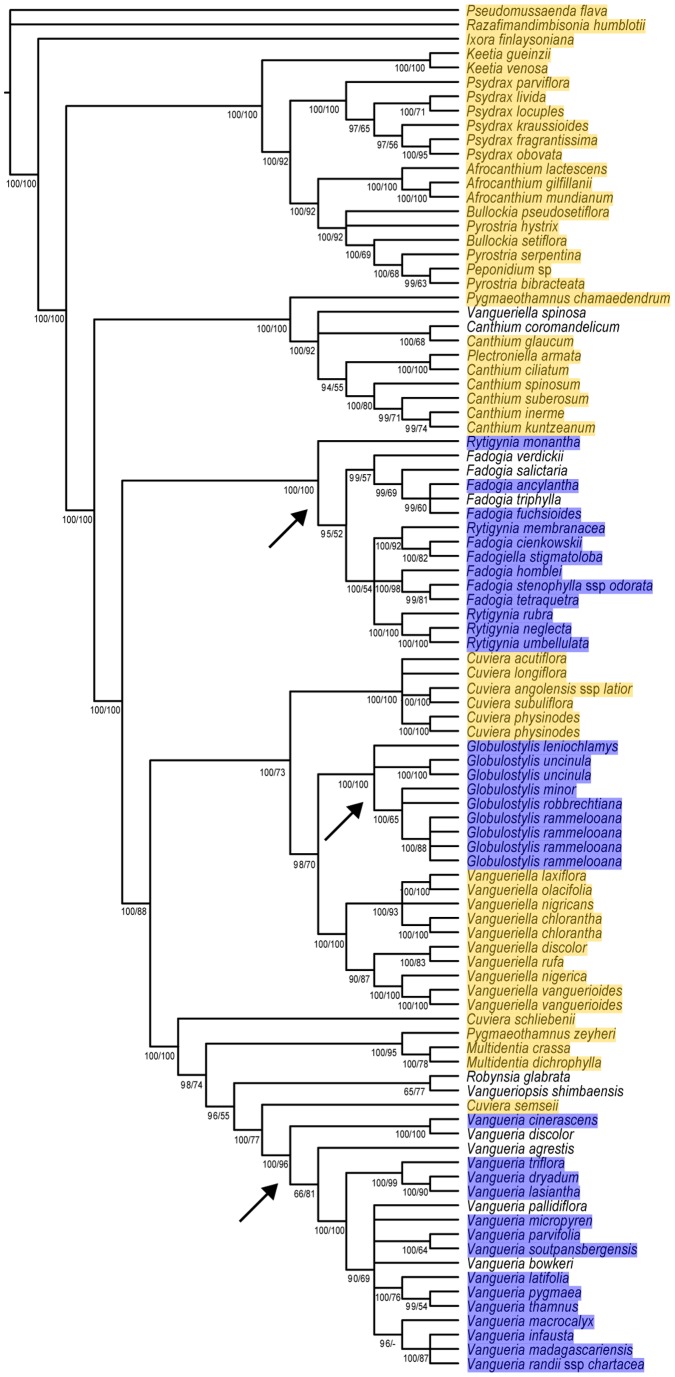
Phylogenetic tree of *Vanguerieae* with presence (blue) and absence (yellow) of endophytic *Burkholderia* bacteria. This phylogenetic dendrogram of the plant tribe *Vanguerieae* is adapted from Verstraete et al. [11]. Bacterial endosymbiosis is found in three groups of the tribe (arrows). Four species were not investigated because we did not have the specimens, and for seven other species we did not find *Burkholderia* endophytes although their phylogenetic position suggests so (not coloured). Bayesian posterior probabilities/bootstrap values are indicated below the branches.

The presence of *Burkholderia* endophytes is limited to five genera of the tribe *Vanguerieae*: *Fadogia*, *Fadogiella*, *Globulostylis*, *Rytigynia* and *Vangueria*. The basal taxa of the tribe (*Afrocanthium*, *Bullockia*, *Keetia*, *Peponidium*, *Psydrax*, *Pyrostria*) and the genera *Canthium* and *Plectroniella* are not found to harbour endophytes in their leaves (Fig. 2). The genera *Cuviera*, *Multidentia*, *Pygmaeothamnus* and *Vangueriella* also lack endophytes. All the species that do not possess endophytic bacteria were re-examined carefully using several biological and technical replicates (78 specimens of 47 species). Visually indicating the presence and absence of endophytic *Burkholderia* on the phylogenetic tree reveals that the association between plants and bacteria seems to occur only in three groups of the tribe *Vanguerieae*: the *Fadogia/Rytigynia* group, *Globulostylis* and *Vangueria* (Fig. 2). For seven species, which are suspected to hold *Burkholderia* endosymbionts because of their phylogenetic position, we were not able to detect endophytes. These provisional negative results were obtained from investigating herbarium specimens for which we only had one replicate. This prevented us from supporting the aberrant observations in these particular plant species.

**Figure 3:**
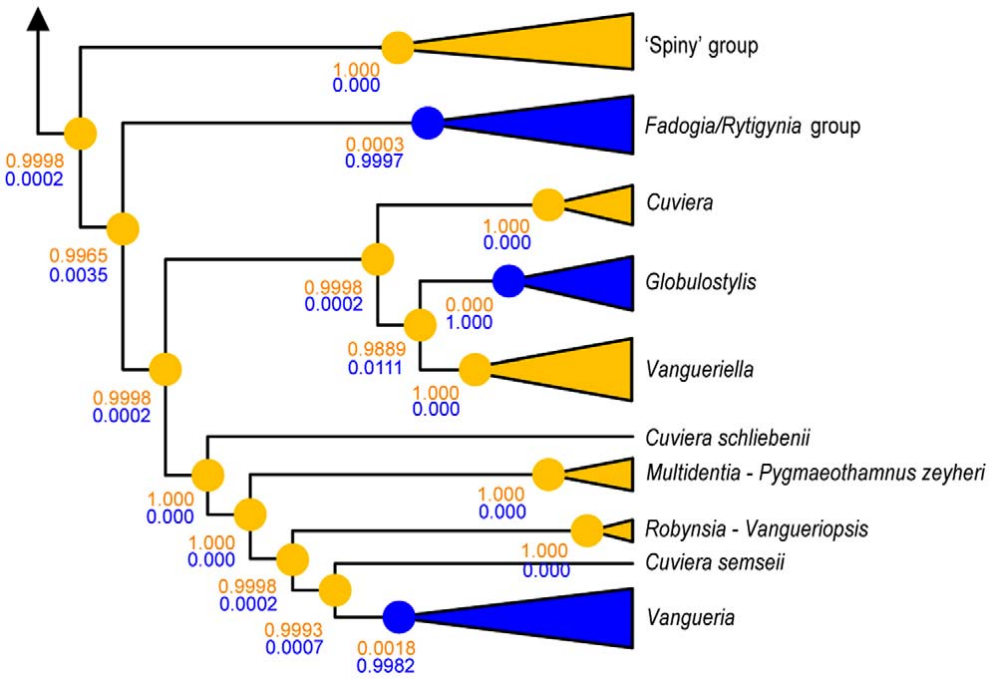
Optimization of the *Vanguerieae*-*Burkholderia* interaction on a simplified representation of the host plant tree. In *Vanguerieae*, the association with *Burkholderia* endophytes has occurred three times independently (blue clades). The circles are pie charts that represent the chance of presence and absence of endophytes per node. However, because the Bayesian posterior probabilities are so high, they are or completely yellow (absence of endophytes) or completely blue (presence of endophytes). Support values for either absence (yellow) or presence (blue) are indicated below the nodes.

The presence of *Burkholderia* endophytes was plotted on the phylogenetic tree of the plants and the statistical support for the occurrence of the interaction was calculated. As already suggested by figure 2, the *Vanguerieae*-*Burkholderia* association has emerged three times independently, and this is well supported statistically (Fig. 3).

**Figure 4:**
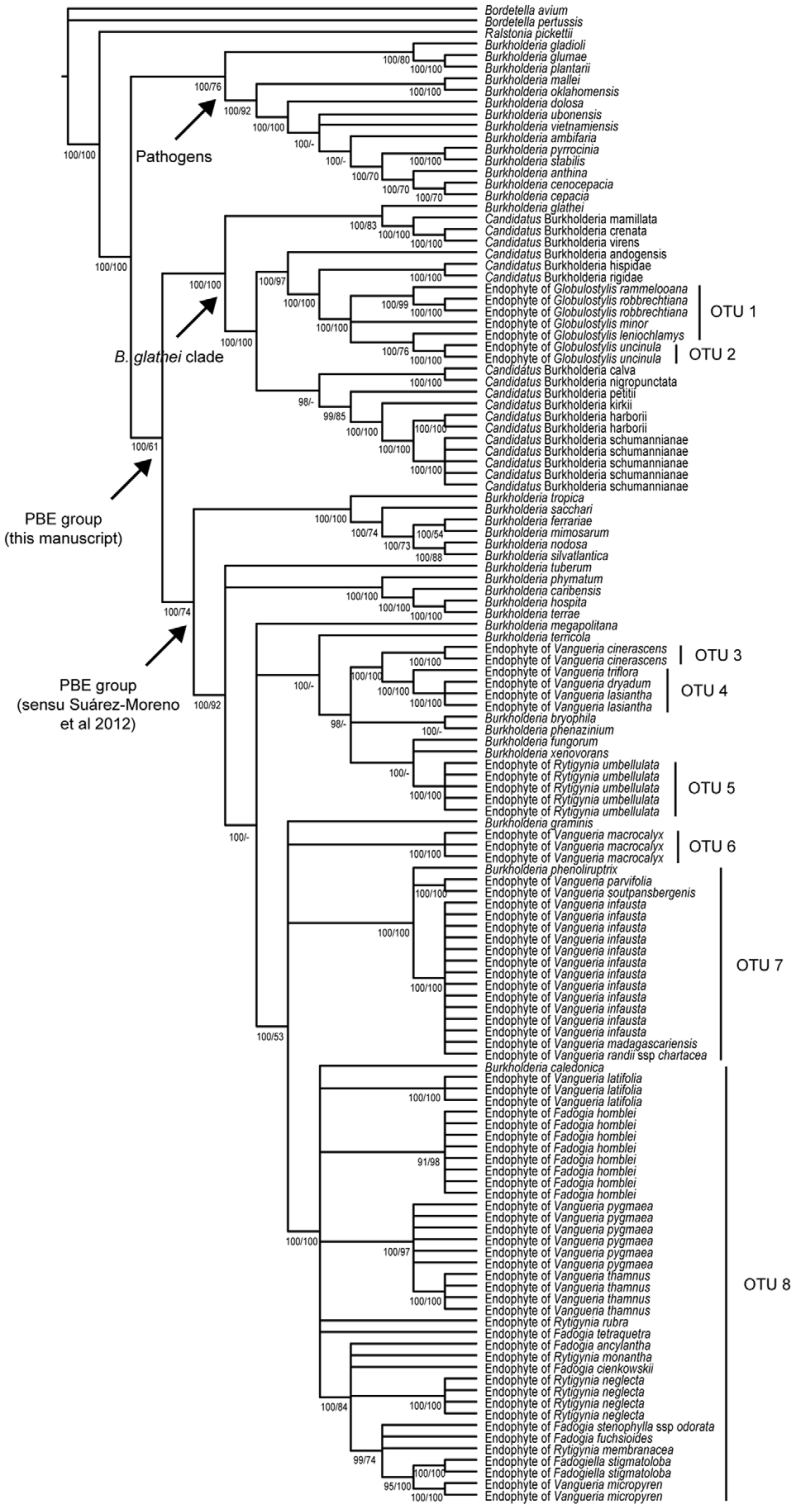
*Burkholderia* tree with the phylogenetic position of the newly discovered endophytes. This phylogenetic dendrogram is based on the concatenated dataset of the 16S rRNA gene, *gyrB* gene and *recA* gene. Several clades are evident: the pathogenic group, a clade with *B. glathei* and related endophytes, and the plant-associated beneficial and environmental (PBE) group sensu Suárez-Moreno et al. (arrows). Most of the new endophytes fall in the latter group, except for the bacteria in *Globulostylis*, which are more related to the previously described *Candidatus* species. We therefore combine the *B. glathei* clade with the PBE group sensu Suárez-Moreno et al., to form a broadly defined PBE group. Bayesian posterior probabilities/bootstrap values are indicated below the branches.

Identification of the bacteria was performed using the standard method of comparing the sequence similarity of the 16S rRNA gene region [19]. Additional support for the relationship is obtained through a combined phylogenetic analysis of three molecular markers (16S rRNA gene, *gyrB* gene and *recA* gene). Information on the bacteria and their accession numbers can be found in Table S2. The preliminary identity of the endophytes was established using BLAST searches on GenBank, and this confirmed a relationship to the genus *Burkholderia*. Many host plant species were investigated using several different specimens to corroborate and support the results (Table S1). These biological replicates point to the presence of one bacterial partner per plant species, because in all investigated specimens of a particular plant species the same endophyte was found. Every individual of the same plant species thus seems to harbour only one type of bacteria (although this bacteria species might occur elsewhere). We defined eight OTUs based on a 16S rRNA gene sequence similarity higher than 99%. The endophytes of 37 specimens of 15 different plant species are shown to be closely related to *B. caledonica* (Fig. 4 OTU 8). The similarity ranges from 99.2% to 100%. Sixteen specimens of 5 other plant species seem to have endophytes that are more related to *B. phenoliruptrix*, with a similarity of 99.4% (Fig. 4 OTU 7). In three plant species (viz. *Vangueria dryadum*, *V. lasiantha* and *V. triflora*) the endophytic bacteria exhibit a similarity of their 16S rRNA gene sequence from 99.8% tot 100% and can therefore be considered as identical (Fig. 4 OTU 4). However, their DNA sequences do not correspond to any previously described bacterial species. The bacteria of OTU 3, 5 and 6 do not show a high similarity with a so far described and recognized *Burkholderia* species (<98% similarity). All newly identified endophytes group more or less together, with the exception of the ones in *Globulostylis*, which are more related to *Burkholderia* found in leaf galls (Fig. 4 OTU 1 and 2). Noteworthy is that none of the *Globulostylis* species has visible galls on the leaves, which would suggest a closer relationship to the plant-associated beneficial and environmental group. Because of this result, we completely re-examined the *Globulostylis* species by extracted new bacterial DNA from different leaves and by sequencing the three DNA markers over again, but the analysis yielded the same result.

## Discussion

Recently, the focus of *Burkholderia* research has shifted from pathogenic species to environmental and plant-associated species [20]–[21]. However, these studies strongly focus on legume-nodulating bacteria and they are apparently unaware of the fact that *Burkholderia* bacteria also have been discovered in close relationship with *Rubiaceae* and *Primulaceae* plants [5]–[9] [22]–[25]. These *Burkholderia* endophytes were not found in the roots, but they were found in clearly visible galls on the leaf blades of some tropical plants. Our research focuses on this subject of bacterial leaf endosymbiosis and shows that there is more to symbiotic ß-proteobacteria than just legumes.

In our study of the *Vanguerieae* tribe (*Rubiaceae*), endophytic *Burkholderia* bacteria were found inside the leaves of many representatives. Previous studies in *Rubiaceae* showed the endophytic lifestyle of the endosymbionts by investigating specialized leaf galls [5] or by cultivating the endophytes on agar plates after surface sterilization with ethanol and sodium hypochlorite [9]. By using scanning electron microscopy we were able to confirm that the bacteria found here are true endophytes and occur between the mesophyll cells of the leaves (Fig. 1E, F). None of the investigated specimens shows any external sign of infection (Fig. 1G), which is in contrast with previously discovered *Burkholderia* endophytes of other rubiaceous genera where visible galls are found in the leaf blades ([8]: their Fig. 3). The presence of endophytes is however limited to five genera: *Fadogia*, *Fadogiella*, *Globulostylis*, *Rytigynia* and *Vangueria* (Fig. 2). The presence of endophytes is consistent on genus level, which serves as a predictive value for the discovery of new bacteria. *Globulostylis* used to be a subgenus of *Cuviera* but has been reinstated as a genus only recently [11]. This taxonomic change is here corroborated, as presence of bacterial endosymbiosis is now consistent on genus level. This consistency also applies to the absence of *Burkholderia* endophytes: in total 11 genera lack them inside their leaves. One cautionary note on the absence of endophytes: a negative result is a provisional result, as other bacteria might not be detectable with currently available techniques. A clear overview of the endosymbiosis is provided when plotting the presence and absence of endophytic *Burkholderia* on the phylogenetic tree of the host plants. This reveals that the association between *Vanguerieae* plants and *Burkholderia* bacteria seems to have occurred in only three groups of the tribe (Fig. 3 blue clades). Investigation of the endophytes found in leaf galls showed a similar result; the plant-bacteria association occurred there at least four times [5]. This pattern can be explained if we assume that *Burkholderia* leaf symbiosis is a newly obtained and recent feature for the plant. An indication for this is that the interaction is not specialized (same endophyte in multiple hosts) and not obligate (endophyte can be cultivated). Although there are some indications that bacterial leaf symbiosis can be hereditary in nodulated host plants [5], our results indicate that at least for *Vanguerieae* a rather loose interaction exists between host and endophyte. Distinct plant species that are colonized by a same species of endophyte (e.g. *Vangueria infausta* and *V. parvifolia* by OTU 7, Fig. 4) co-occur in the wild and are found in the same habitat, which again argues for a more facultative association between host and endophyte. Furthermore, *Burkholderia* are commonly isolated from the soil [26] and free-living *Burkholderia* are nested within leaf endophyte clades (Fig. 4; [5]), which indicates that exchange between the host and soil niche is reasonable to accept. The actual transfer mechanism and the frequency of these ongoing reinfection events are still unknown and remain to be tested.

The review of Gyaneshwar et al. [20] states that two main clusters within the genus *Burkholderia* occur; one cluster comprises human, animal and plant pathogens, while the second cluster contains non-pathogenic species associated with plants and/or the environment. A second review also noted that non-pathogenic plant-associated bacteria form one single clade of closely related species and they called it the ‘plant-associated beneficial and environmental (PBE) group’ [21]. Most studies on plant-associated *Burkholderia* have concentrated on *Mimosa* spp. (*Fabaceae*) and have found that the symbionts are more related to ‘environmental’ rather than ‘pathogenic’ *Burkholderia* [27]. These ß-rhizobia are able to fix nitrogen and are therefore beneficial to their host plants [28]. Nitrogenase activity and the presence of *nifH* genes in *Burkholderia* isolates from the rhizosphere of tomato plants demonstrated their diazotrophic abilities [29]. However, not all *Burkholderia* endophytes seem to be capable of fixing nitrogen, as demonstrated by the lack of genetic evidence for diazotrophy in the leaf symbiont of *Psychotria kirkii* [10].

In our study, all newly identified endophytes from OTU 3 to 8 clearly fall in the PBE group sensu Suárez-Moreno et al. (Fig. 4). This phylogenetic placement is not surprising, because all other plant-associated *Burkholderia* are grouped here and because other endophytes of *Vanguerieae* have been identified earlier [9]. When observing the phylogenetic tree more in detail, it is clear that every specimen of the same plant species harbours only one species of *Burkholderia* (Fig. 4). We conclude this based on previous studies in *Rubiaceae* [5] [9], the different biological replicates of one host species that yield the same result, and the primer designed specifically for *Burkholderia*. Although the presence of a second endosymbiont occurring in low density can never be ruled out, we are confident that *Burkholderia* is the main endophyte and that there is only a single species present. However, this one species of bacteria is not always limited to one plant species; e.g. based on 16S rRNA gene all bacteria in OTU 8 are considered to be *B. caledonica*, but these endophytes are found in many different plant species (Fig. 4). This could suggest that the host plant is somehow able to select for a specific bacteria species that could be beneficial to its fitness. The combined analysis of the three DNA markers, however, shows host specificity of the bacteria at population level (Fig. 4). The different specimens from one plant species group together, which means there is one specific group of bacteria or a ‘bacterial population’ in one plant species; e.g. endophytes of *Rytigynia neglecta* in OTU 8. This has already been observed for the endophytes of the genera *Fadogia* and *Vangueria* [9]. Figure 4 also shows that endophytes of related plant species sometimes cluster together. The bacteria in *Vangueria madagascariensis* and *V. randii* for example, are closely related to the ones of *V. infausta*. This could point to some degree of coevolution, but there could be another explanation: the mutual relationships between these plants are uncertain and these different plant species might be varieties of a single species. Should this be the case, it provides a clear example of the possible usefulness and application of bacterial leaf endosymbiosis in plant taxonomy. It should also be noted that these closely related plant species need similar environmental conditions or occur in the same geographical region. External infections from soil bacteria could then explain why they share the same endophyte. This open plant-bacteria interaction was also postulated for nodulating *Burkholderia* symbionts [5].

The *Burkholderia* endophytes found in the plant genus *Globulostylis* are aberrant and are more closely related to the nodulating *Rubiaceae* endophytes (Fig. 4 OTU 1 and 2). This finding is peculiar because all *Vanguerieae* endophytes so far are member of the PBE group (sensu Suárez-Moreno et al.) and the *Globulostylis* species do not have visible bacterial galls in their leaf blades. These results are, however, corroborated by the observation of non-nodulating endophytes in *Psychotria* species, the genus that is especially known for its leaf galls [23]. The closest relative of all these nodulating endophytes is *B. glathei*, a free-living soil bacterium that is not considered to be part of the PBE group according to Suárez-Moreno et al.; instead it is placed in the pathogenic *Burkholderia* clade. Our analysis differs from this study in the fact that *B. glathei* and the related endophytes clearly form a separate group within the genus *Burkholderia*. Three distinct groups can be seen: the first group corresponds to the pathogenic group, a second group contains *B. glathei* with related endophytes, and the last clade is the PBE group sensu Suárez-Moreno et al. (Fig. 4). However, it seems logical to assign the entire *B. glathei* clade to a broadly defined PBE group, as it concerns an environmental bacterium and several endophytic *Candidatus* species. By doing so, the PBE group is greatly expanded and better reflects the actual diversity of the plant-associated bacteria.

In summary, symbiotic ß-proteobacteria of the genus *Burkholderia* have been discovered in plants formerly not implicated in endosymbiosis. These findings suggest a wider occurrence of host-endosymbiont interactions. Plotting the presence and absence on the phylogenetic tree of the host plants revealed that the *Vanguerieae*-*Burkholderia* association has emerged three times independently. This suggests a recent and facultative plant-bacteria interaction. When bacteria are found in one species of a particular genus, other representatives of the same genus also have endophytes. On species level every plant has its unique endophyte, although this bacterial species may occur elsewhere. Two main clades were recognized in *Burkholderia* until now and most of the newly discovered endophytes are part of the PBE group sensu Suárez-Moreno et al. However, *B. glathei*, formerly in the pathogenic group, together with bacteria found in leaf galls and some non-nodulating endophytes form a third clade within the genus. We therefore propose to include this *B. glathei* clade in a newly defined and larger PBE group.

## Supporting Information

Table S1 **List of all host plants that were investigated for bacterial endosymbiosis.** Including vouchers, origin and presence (+), absence (−) or unknown status (?) of *Burkholderia* endophytes. In total 165 specimens of 89 species were checked: 76 specimens of 31 species harbour endophytes, while 78 specimens of 47 species lack them. The presence of endophytes was not investigated in 11 species. Herbarium abbreviations are according to the Index Herbariorum.
(DOC)

Table S2 **Detailed list of the endophytes found in the** ***Vanguerieae*** **tribe.** Information on taxon, voucher and GenBank accession numbers for 16S rRNA gene, *gyrB* and *recA*. -, no sequence.
(DOC)
