# Supplementary material for: Symbiotic ß-Proteobacteria beyond Legumes: Burkholderia in Rubiaceae
Source: PLoS One. 2013 Jan 25;8(1):e55260. doi: 10.1371/journal.pone.0055260 (PMC3555867; doi:10.1371/journal.pone.0055260)
Supplement: Table S1 — List of all host plants that were investigated for bacterial endosymbiosis. Including vouchers, origin and presence (+), absence (−) or unknown status (?) of Burkholderia endophytes. In total 165 specimens of 89 species were checked: 76 specimens of 31 species harbour endophytes, while 78 specimens of 47 species lack them. The presence of endophytes was not investigated in 11 species. Herbarium abbreviations are according to the Index Herbariorum. (DOC) [file pone.0055260.s001.doc]

| Taxon | Voucher | Origin | Bacteria |
| --- | --- | --- | --- |
| *Afrocanthium gilfillanii* | Lemaire & Verstraete 32 (BR) | South Africa | - |
| *Afrocanthium lactescens* | Kuchar 23003 (BR) |  | - |
| *Afrocanthium mundianum* | Lemaire & Verstraete 33 (BR) | South Africa | - |
| *Afrocanthium mundianum* | Lemaire & Verstraete 84 (BR) | South Africa | - |
| *Bullockia pseudosetiflora* | Bidgood et al 4959 (BR) |  | - |
| *Bullockia setiflora* | Burrows & Burrows 10350 (BNRH) | Swaziland | - |
| *Bullockia setiflora* | Lemaire & Verstraete 161 (BR) | South Africa | - |
| *Bullockia setiflora* | Lemaire & Verstraete 197 (BR) | South Africa | - |
| *Canthium ciliatum* | Burrows 6259 (BNRH) | South Africa | - |
| *Canthium ciliatum* | Lemaire & Verstraete 122 (BR) | South Africa | - |
| *Canthium ciliatum* | Lemaire & Verstraete 281 (BR) | South Africa | - |
| *Canthium coromandelicum* | Andreasen 36 (UPS) |  | ? |
| *Canthium glaucum* | Kuchar 17410 (BR) |  | - |
| *Canthium inerme* | Lemaire & Verstraete 235 (BR) | South Africa | - |
| *Canthium kuntzeanum* | Lemaire & Verstraete 241 (BR) | South Africa | - |
| *Canthium kuntzeanum* | Lemaire & Verstraete 250 (BR) | South Africa | - |
| *Canthium spinosum* | Lemaire & Verstraete 187 (BR) | South Africa | - |
| *Canthium suberosum* | Burrows & Burrows 9858 (BNRH) | South Africa | - |
| *Cuviera acutiflora* | Dessein et al 2809 (BR) | Cameroon | - |
| *Cuviera angolensis* ssp *latior* | Amsini 97 (BR) | D.R.Congo | - |
| *Cuviera longiflora* | Dessein et al 2713 (BR) | Cameroon | - |
| *Cuviera physinodes* | Chatrou 572 (BR) | Cameroon | - |
| *Cuviera physinodes* | Dessein et al 1416 (BR) | Cameroon | - |
| *Cuviera schliebenii* | Burrows & Burrows 9839 (BNRH) | Mozambique | - |
| *Cuviera semseii* | Burrows & Burrows 11303 (BNRH) | Mozambique | - |
| *Cuviera subuliflora* | Dessein et al 2004 (BR) | Gabon | - |
| *Cuviera subuliflora* | Dessein et al 2864 (BR) | Cameroon | - |
| *Fadogia ancylantha* | Dessein et al 1101 (BR) | Zambia | + |
| *Fadogia cienkowskii* | Dessein et al 258 (BR) | Zambia | + |
| *Fadogia fuchsioides* | Dessein et al 1083 (BR) | Zambia | + |
| *Fadogia homblei* | 20101674-73 (NBGB) | South Africa | + |
| *Fadogia homblei* | Lemaire & Verstraete 3 (BR) | South Africa | + |
| *Fadogia homblei* | Lemaire & Verstraete 9 (BR) | South Africa | + |
| *Fadogia homblei* | Lemaire & Verstraete 22 (BR) | South Africa | + |
| *Fadogia homblei* | Lemaire & Verstraete 30 (BR) | South Africa | + |
| *Fadogia homblei* | Lemaire & Verstraete 50 (BR) | South Africa | + |
| *Fadogia homblei* | Lemaire & Verstraete 57 (BR) | South Africa | + |
| *Fadogia homblei* | Lemaire & Verstraete 292 (BR) | South Africa | + |
| *Fadogia salictaria* | Malaisse 13490 (BR) |  | ? |
| *Fadogia stenophylla* ssp *odorata* | Lovett 2267 (BR) | Tanzania | + |
| *Fadogia tetraquetra* | Lemaire & Verstraete 223 (BR) | South Africa | + |
| *Fadogia triphylla* | Dessein et al 226 (BR) | Zambia | ? |
| *Fadogia verdickii* | Schaijes 1502 (BR) |  | ? |
| *Fadogiella stigmatoloba* | Dessein et al 337 (BR) | Zambia | + |
| *Fadogiella stigmatoloba* | Gillett 17403 (BR) | Tanzania | + |
| *Globulostylis leniochlamys* | Dessein et al 1448 (BR) | Cameroon | + |
| *Globulostylis minor* | Dessein et al 2876 (BR) | Cameroon | + |
| *Globulostylis rammelooana* | Dessein et al 1546 (BR) | Cameroon | + |
| *Globulostylis rammelooana* | Sonké et al 4650 (BR) | Cameroon | + |
| *Globulostylis rammelooana* | Sonké et al 4671 (BR) | Cameroon | + |
| *Globulostylis rammelooana* | Sonké et al 4711 (BR) | Cameroon | + |
| *Globulostylis robbrechtiana* | Sonké et al 4868 (BR) | Cameroon | + |
| *Globulostylis uncinula* | Dessein et al 2116 (BR) | Gabon | + |
| *Globulostylis uncinula* | Dessein et al 2337 (BR) | Gabon | + |
| *Ixora finlaysoniana* | Van Caekenberghe 54 (BR) |  | - |
| *Keetia gueinzii* | Lemaire & Verstraete 97 (BR) | South Africa | - |
| *Keetia gueinzii* | Lemaire & Verstraete 127 (BR) | South Africa | - |
| *Keetia gueinzii* | Lemaire & Verstraete 210 (BR) | South Africa | - |
| *Keetia gueinzii* | Lemaire & Verstraete 225 (BR) | South Africa | - |
| *Keetia gueinzii* | Lemaire & Verstraete 300 (BR) | South Africa | - |
| *Keetia venosa* | Burrows & Burrows 10007 (BNRH) | Mozambique | - |
| *Keetia venosa* | Dessein et al 1261 (BR) | Zambia | - |
| *Multidentia crassa* | Dessein et al 1085 (BR) | Zambia | - |
| *Multidentia crassa* | Dessein et al 1236 (BR) | Zambia | - |
| *Multidentia dichrophylla* | Dessein et al 2949 (BR) | Cameroon | - |
| *Peponidium* sp. | De Block et al 2487 (BR) | Madagascar | - |
| *Plectroniella armata* | Lemaire & Verstraete 150 (BR) | South Africa | - |
| *Plectroniella armata* | Lemaire & Verstraete 165 (BR) | South Africa | - |
| *Plectroniella armata* | Lemaire & Verstraete 174 (BR) | South Africa | - |
| *Plectroniella armata* | Lemaire & Verstraete 255 (BR) | South Africa | - |
| *Plectroniella armata* | Lemaire & Verstraete 266 (BR) | South Africa | - |
| *Pseudomussaenda flava* | Van Caekenberghe 60 (BR) |  | - |
| *Psydrax fragrantissima* | Lemaire & Verstraete 163 (BR) | South Africa | - |
| *Psydrax kraussioides* | Dessein et al 1285 (BR) | Zambia | - |
| *Psydrax kraussioides* | Dessein et al 2979 (BR) | Cameroon | - |
| *Psydrax livida* | Lemaire & Verstraete 14 (BR) | South Africa | - |
| *Psydrax livida* | Lemaire & Verstraete 261 (BR) | South Africa | - |
| *Psydrax locuples* | Lemaire & Verstraete 287 (BR) | South Africa | - |
| *Psydrax obovata* | Lemaire & Verstraete 188 (BR) | South Africa | - |
| *Psydrax obovata* | Lemaire & Verstraete 224 (BR) | South Africa | - |
| *Psydrax parviflora* | Dessein et al 1345 (BR) |  | - |
| *Pygmaeothamnus chamaedendrum* | Lemaire & Verstraete 142 (BR) | South Africa | - |
| *Pygmaeothamnus zeyheri* | Dessein et al 326 (BR) | Zambia | - |
| *Pygmaeothamnus zeyheri* | Lemaire & Verstraete 2 (BR) | South Africa | - |
| *Pygmaeothamnus zeyheri* | Lemaire & Verstraete 58 (BR) | South Africa | - |
| *Pygmaeothamnus zeyheri* var *rogersii* | Dessein et al 663 (BR) | Zambia | - |
| *Pyrostria bibracteata* | Burrows & Burrows 10784 (BNRH) | Mozambique | - |
| *Pyrostria hystrix* | Lemaire & Verstraete 92 (BR) | South Africa | - |
| *Pyrostria hystrix* | Lemaire & Verstraete 270 (BR) | South Africa | - |
| *Pyrostria serpentina* | De Block et al 2423 (BR) | Madagascar | - |
| *Razafimandimbisonia humblotii* | Tosh et al 263 (BR) | Madagascar | - |
| *Robynsia glabrata* | Hall & Amponsah 46545 (K) |  | ? |
| *Rytigynia membranacea* | Lachenaud et al 739 (BR) | Cameroon | + |
| *Rytigynia monantha* | Niyongabo 53 (BR) | Burundi | + |
| *Rytigynia neglecta* | Dessein et al 2958 (BR) | Cameroon | + |
| *Rytigynia neglecta* | Dessein et al 2961 (BR) | Cameroon | + |
| *Rytigynia neglecta* | Dessein et al 3053 (BR) | Cameroon | + |
| *Rytigynia neglecta* | Dessein et al 3056 (BR) | Cameroon | + |
| *Rytigynia rubra* | Dessein et al 2541 (BR) | Cameroon | + |
| *Rytigynia umbellulata* | Dessein et al 2940 (BR) | Cameroon | + |
| *Rytigynia umbellulata* | Dessein et al 2944 (BR) | Cameroon | + |
| *Rytigynia umbellulata* | Dessein et al 3087 (BR) | Cameroon | + |
| *Rytigynia umbellulata* | Dessein et al 3123 (BR) | Cameroon | + |
| *Rytigynia umbellulata* | Lachenaud et al 852 (BR) | Cameroon | + |
| *Vangueria agrestis* | Lejoly 82/390 (BR) |  | ? |
| *Vangueria bowkeri* | Lemaire & Verstraete 233 (BR) | South Africa | ? |
| *Vangueria cinerascens* | Dessein et al 202 (BR) | Zambia | + |
| *Vangueria cinerascens* | Dessein et al 443 (BR) | Zambia | + |
| *Vangueria discolor* | Bidgood et al 6094 (BR) |  | ? |
| *Vangueria dryadum* | Lemaire & Verstraete 289 (BR) | South Africa | + |
| *Vangueria infausta* | Dessein et al 879A (BR) | Zambia | + |
| *Vangueria infausta* | Dessein et al 879B (BR) | Zambia | + |
| *Vangueria infausta* | Dessein et al 879C (BR) | Zambia | + |
| *Vangueria infausta* | Dessein et al 879D (BR) | Zambia | + |
| *Vangueria infausta* | Lemaire & Verstraete 13 (BR) | South Africa | + |
| *Vangueria infausta* | Lemaire & Verstraete 21 (BR) | South Africa | + |
| *Vangueria infausta* | Lemaire & Verstraete 31 (BR) | South Africa | + |
| *Vangueria infausta* | Lemaire & Verstraete 38 (BR) | South Africa | + |
| *Vangueria infausta* | Lemaire & Verstraete 51 (BR) | South Africa | + |
| *Vangueria infausta* | Lemaire & Verstraete 61 (BR) | South Africa | + |
| *Vangueria infausta* | Lemaire & Verstraete 207 (BR) | South Africa | + |
| *Vangueria infausta* | Lemaire & Verstraete 279 (BR) | South Africa | + |
| *Vangueria lasiantha* | Lemaire & Verstraete 209 (BR) | South Africa | + |
| *Vangueria lasiantha* | Lemaire & Verstraete 269 (BR) | South Africa | + |
| *Vangueria latifolia* | Lemaire & Verstraete 69 (BR) | South Africa | + |
| *Vangueria latifolia* | Lemaire & Verstraete 74 (BR) | South Africa | + |
| *Vangueria latifolia* | Lemaire & Verstraete 141 (BR) | South Africa | + |
| *Vangueria macrocalyx* | Lemaire & Verstraete 68 (BR) | South Africa | + |
| *Vangueria macrocalyx* | Lemaire & Verstraete 71 (BR) | South Africa | + |
| *Vangueria macrocalyx* | Lemaire & Verstraete 114 (BR) | South Africa | + |
| *Vangueria madagascariensis* | Lemaire & Verstraete 189 (BR) | South Africa | + |
| *Vangueria micropyren* | Dessein et al 253 (BR) | Zambia | + |
| *Vangueria micropyren* | Dessein et al 929 (BR) | Zambia | + |
| *Vangueria pallidiflora* | Ntemi Sallu et al 309 (BR) |  | ? |
| *Vangueria parvifolia* | Lemaire & Verstraete 265 (BR) | South Africa | + |
| *Vangueria pygmaea* | Dessein et al 666 (BR) | Zambia | + |
| *Vangueria pygmaea* | Dessein et al 726 (BR) | Zambia | + |
| *Vangueria pygmaea* | Lemaire & Verstraete 26A (BR) | South Africa | + |
| *Vangueria pygmaea* | Lemaire & Verstraete 26B (BR) | South Africa | + |
| *Vangueria pygmaea* | Lemaire & Verstraete 28 (BR) | South Africa | + |
| *Vangueria pygmaea* | Lemaire & Verstraete 42 (BR) | South Africa | + |
| *Vangueria randii* ssp *chartacea* | Lemaire & Verstraete 98 (BR) | South Africa | + |
| *Vangueria soutpansbergensis* | Lemaire & Verstraete 285 (BR) | South Africa | + |
| *Vangueria thamnus* | Bester 10538 (PRE) | South Africa | + |
| *Vangueria thamnus* | Lemaire & Verstraete 25A (BR) | South Africa | + |
| *Vangueria thamnus* | Lemaire & Verstraete 25B (BR) | South Africa | + |
| *Vangueria thamnus* | Steyn 1835 (PRE) | South Africa | + |
| *Vangueria triflora* | Lemaire & Verstraete 53 (BR) | South Africa | + |
| *Vangueriella chlorantha* | Dessein et al 1632 (BR) | Cameroon | - |
| *Vangueriella chlorantha* | Dessein et al 1654 (BR) | Cameroon | - |
| *Vangueriella chlorantha* | Dessein et al 3180 (BR) | Cameroon | - |
| *Vangueriella chlorantha* | Lachenaud et al 662 (BR) | Cameroon | - |
| *Vangueriella chlorantha* | Lachenaud et al 810 (BR) | Cameroon | - |
| *Vangueriella chlorantha* | Lachenaud et al 827 (BR) | Cameroon | - |
| *Vangueriella discolor* | Jongkind 7887 (WAG) |  | - |
| *Vangueriella laxiflora* | Lachenaud et al 689 (BR) | Cameroon | - |
| *Vangueriella nigerica* | Jongkind 3089 (WAG) |  | - |
| *Vangueriella nigricans* | Dessein et al 1574 (BR) | Cameroon | - |
| *Vangueriella olacifolia* | Dessein et al 2415 (BR) | Gabon | - |
| *Vangueriella rufa* | Dessein et al 2310 (BR) | Gabon | - |
| *Vangueriella rufa* | Lachenaud et al 1119 (BR) | Cameroon | - |
| *Vangueriella spinosa* | Merello et al 1494 (K) |  | ? |
| *Vangueriella vanguerioides* | Jongkind 7855 (WAG) |  | - |
| *Vangueriella vanguerioides* | Kpadeyeah 10 (WAG) |  | - |
| *Vangueriopsis shimbaensis* | Luke 8316 (UPS) |  | ? |
