# Supplementary material for: Symbiotic ß-Proteobacteria beyond Legumes: Burkholderia in Rubiaceae
Source: PLoS One. 2013 Jan 25;8(1):e55260. doi: 10.1371/journal.pone.0055260 (PMC3555867; doi:10.1371/journal.pone.0055260)
Supplement: Table S2 — Detailed list of the endophytes found in the Vanguerieae tribe. Information on taxon, voucher and GenBank accession numbers for 16S rRNA gene, gyrB and recA. -, no sequence. (DOC) [file pone.0055260.s002.doc]

| Taxon | Voucher | 16S | *gyrB* | *recA* |
| --- | --- | --- | --- | --- |
| *Bordetella avium* | 197N | BAVs02 | BAV3410 | BAV2309 |
| *Bordetella pertussis* | Tohama I | BPr01 | BP0489 | BP2546 |
| *Ralstonia picketii* | 12J | NC010678 | NC010682 | NC010682 |
| *Burkholderia ambifaria* | LMG 19182 | HQ849072 | HQ849186 | HQ849130 |
| *Burkholderia anthina* | LMG 20980 | HQ849074 | HQ849187 | HQ849132 |
| *Burkholderia bryophila* | LMG 23644 | HQ849075 | HQ849188 | HQ849133 |
| *Burkholderia caledonica* | LMG 19076 | HQ849076 | HQ849189 | HQ849134 |
| *Burkholderia caribensis* | LMG 18531 | HQ849077 | HQ849190 | HQ849135 |
| *Burkholderia cenocepacia* | LMG 16656 | AF148556 | DQ124427 | AY951880 |
| *Burkholderia cepacia* | LMG 1222 | HQ849078 | HQ849191 | JF295011 |
| *Burkholderia dolosa* | LMG 18943 | HQ849079 | HQ849192 | HQ849136 |
| *Burkholderia ferrariae* | LMG 23612 | HQ849080 | HQ849193 | HQ849137 |
| *Burkholderia fungorum* | LMG 16225 | HQ849081 | HQ849194 | HQ849138 |
| *Burkholderia gladioli* pv *gladioli* | LMG 2216 | HQ849083 | HQ849196 | HQ849140 |
| *Burkholderia glathei* | LMG 14190 | HQ849084 | HQ849197 | HQ849141 |
| *Burkholderia glumae* | LMG 2196 | HQ849085 | HQ849198 | HQ849142 |
| *Burkholderia graminis* | LMG 18924 | HQ849086 | HQ849199 | HQ849143 |
| *Burkholderia hospita* | LMG 20598 | HQ849087 | HQ849200 | HQ849144 |
| *Burkholderia mallei* | NCTC 10229 | NC008835 | NC008836 | NC008836 |
| *Burkholderia megapolitana* | LMG 23650 | AM489502 | - | - |
| *Burkholderia mimosarum* | LMG 23256 | HQ849089 | HQ849202 | HQ849146 |
| *Burkholderia nodosa* | LMG 23741 | HQ849091 | HQ849204 | HQ849147 |
| *Burkholderia oklahomensis* | LMG 23618 | HQ849092 | HQ849205 | HQ849148 |
| *Burkholderia phenazinium* | LMG 2247 | HQ849093 | HQ849206 | HQ849149 |
| *Burkholderia phenoliruptrix* | LMG 22037 | HQ849094 | HQ849207 | HQ849150 |
| *Burkholderia phymatum* | LMG 21445 | HQ849095 | HQ849208 | HQ849151 |
| *Burkholderia plantarii* | LMG 9035 | HQ849098 | HQ849210 | HQ849153 |
| *Burkholderia pyrrocinia* | LMG 14191 | HQ849099 | HQ849211 | HQ849155 |
| *Burkholderia sacchari* | LMG 19450 | HQ849100 | HQ849212 | HQ849156 |
| *Burkholderia silvatlantica* | LMG 23149 | HQ849102 | HQ849213 | HQ849157 |
| *Burkholderia stabilis* | LMG 14294 | HQ849103 | JF295010 | HQ849159 |
| *Burkholderia terrae* | LMG 23368 | AB201285 | - | - |
| *Burkholderia terricola* | LMG 20594 | HQ849104 | HQ849215 | HQ849160 |
| *Burkholderia tropica* | LMG 22274 | HQ849105 | HQ849216 | HQ849161 |
| *Burkholderia tuberum* | LMG 21444 | HQ849106 | HQ849217 | HQ849162 |
| *Burkholderia ubonensis* | LMG 20358 | EU024179 | EU024234 | AY780511 |
| *Burkholderia vietnamiensis* | LMG 10929 | HQ849107 | HQ849218 | HQ849163 |
| *Burkholderia xenovorans* | LMG 21463 | HQ849108 | HQ849219 | HQ849164 |
| Candidatus *Burkholderia andongensis* | Dessein et al 1097 (BR) | JF916921 | JF916905 | JF916915 |
| Candidatus *Burkholderia calva* | 19640306 (NBGB) | HQ849117 | HQ849227 | HQ849173 |
| Candidatus *Burkholderia crenata* | 19073685 (NBGB) | JF416282 | JF416288 | - |
| Candidatus *Burkholderia harborii* | Lemaire & Verstraete 49A | JF265202 | JF265179 | JF265225 |
| Candidatus *Burkholderia harborii* | Lemaire & Verstraete 49B | JF265203 | JF265180 | JF265226 |
| Candidatus *Burkholderia hispidae* | Lachenaud et al 732 (BR) | HQ849123 | HQ849232 | HQ849179 |
| Candidatus *Burkholderia kirkii* | 19536779 (NBGB) | HQ849109 | HQ849220 | HQ849165 |
| Candidatus *Burkholderia mamillata* | 10005023 (NBGB) | JF416284 | JF416290 | - |
| Candidatus *Burkholderia nigropunctata* | Stoffelen et al 13 (BR) | HQ849119 | JF295008 | HQ849175 |
| Candidatus *Burkholderia petitii* | Dessein et al 1512 (BR) | JF916923 | JF916911 | JF916916 |
| Candidatus *Burkholderia rigidae* | Lachenaud et al 877 (BR) | HQ849121 | HQ849230 | HQ849177 |
| Candidatus *Burkholderia schumannianae* | 20019442-57 (NBGB) | HQ849126 | HQ849235 | HQ849182 |
| Candidatus *Burkholderia schumannianae* | 20041430-66 (NBGB) | HQ849127 | HQ849236 | HQ849183 |
| Candidatus *Burkholderia schumannianae* | Dessein et al 1099 (BR) | HQ849124 | HQ849233 | HQ849180 |
| Candidatus *Burkholderia schumannianae* | Dessein et al 1137 (BR) | HQ849125 | HQ849234 | HQ849181 |
| Candidatus *Burkholderia schumannianae* | Lemaire & Verstraete 1 | HQ849128 | HQ849237 | HQ849184 |
| Candidatus *Burkholderia virens* | 20042025 (RBGE) | JF416286 | JF416292 | - |
| Endophyte of *Fadogia ancylantha* | Dessein et al 1101 (BR) | JX064913 | JX065003 | JX065065 |
| Endophyte of *Fadogia cienkowskii* | Dessein et al 258 (BR) | JX064916 | JX065006 | JX065064 |
| Endophyte of *Fadogia fuchsioides* | Dessein et al 1083 (BR) | JX064914 | JX065004 | JX065061 |
| Endophyte of *Fadogia homblei* | 20101674-73 (NBGB) | JF265201 | JF265178 | JF265224 |
| Endophyte of *Fadogia homblei* | Lemaire & Verstraete 3 (BR) | JF265197 | JF265174 | JF265220 |
| Endophyte of *Fadogia homblei* | Lemaire & Verstraete 9 (BR) | JF265200 | JF265177 | JF265223 |
| Endophyte of *Fadogia homblei* | Lemaire & Verstraete 22 (BR) | JF265194 | JF265171 | JF265217 |
| Endophyte of *Fadogia homblei* | Lemaire & Verstraete 30 (BR) | JF265196 | JF265173 | JF265219 |
| Endophyte of *Fadogia homblei* | Lemaire & Verstraete 50 (BR) | JF265198 | JF265175 | JF265221 |
| Endophyte of *Fadogia homblei* | Lemaire & Verstraete 57 (BR) | JF265199 | JF265176 | JF265222 |
| Endophyte of *Fadogia homblei* | Lemaire & Verstraete 292 (BR) | JF265195 | JF265172 | JF265218 |
| Endophyte of *Fadogia stenophylla* ssp *odorata* | Lovett 2267 (BR) | JX064919 | JX065009 | JX065059 |
| Endophyte of *Fadogia tetraquetra* | Lemaire & Verstraete 223 (BR) | JX064920 | JX065010 | JX065055 |
| Endophyte of *Fadogiella stigmatoloba* | Dessein et al 337 (BR) | JX064918 | JX065008 | JX065063 |
| Endophyte of *Fadogiella stigmatoloba* | Gillett 17403 (BR) | JX064917 | JX065007 | JX065062 |
| Endophyte of *Globulostylis leniochlamys* | Dessein et al 1448 (BR) | JX064935 | JX064967 | JX065020 |
| Endophyte of *Globulostylis minor* | Dessein et al 2876 (BR) | JX064931 | JX064968 | JX065021 |
| Endophyte of *Globulostylis rammelooana* | Dessein et al 1546 (BR) | JX064934 | JX064969 | JX065024 |
| Endophyte of *Globulostylis robbrechtiana* | Sonke et al 4868 (BR) | JX064932 | JX064970 | JX065023 |
| Endophyte of *Globulostylis robbrechtiana* | Sonke et al 4872 (BR) | JX064933 | JX064971 | JX065022 |
| Endophyte of *Globulostylis uncinula* | Dessein et al 2116 (BR) | JX064936 | JX064965 | JX065018 |
| Endophyte of *Globulostylis uncinula* | Dessein et al 2337 (BR) | JX064937 | JX064966 | JX065019 |
| Endophyte of *Rytigynia membranacea* | Lachenaud et al 739 (BR) | JX064915 | JX065005 | JX065060 |
| Endophyte of *Rytigynia monantha* | Niyongabo 53 (BR) | JX064912 | JX065002 | JX065066 |
| Endophyte of *Rytigynia neglecta* | Dessein et al 2958 (BR) | JX064924 | JX065013 | JX065067 |
| Endophyte of *Rytigynia neglecta* | Dessein et al 2961 (BR) | JX064922 | JX065012 | JX065068 |
| Endophyte of *Rytigynia neglecta* | Dessein et al 3053 (BR) | JX064925 | JX065014 | JX065069 |
| Endophyte of *Rytigynia neglecta* | Dessein et al 3056 (BR) | JX064923 | JX065015 | JX065070 |
| Endophyte of *Rytigynia rubra* | Dessein et al 2541 (BR) | JX064921 | JX065011 | JX065056 |
| Endophyte of *Rytigynia umbellulata* | Dessein et al 2940 (BR) | JX064927 | JX064974 | JX065026 |
| Endophyte of *Rytigynia umbellulata* | Dessein et al 2944 (BR) | JX064928 | JX064975 | JX065027 |
| Endophyte of *Rytigynia umbellulata* | Dessein et al 3087 (BR) | JX064929 | JX064972 | JX065028 |
| Endophyte of *Rytigynia umbellulata* | Dessein et al 3123 (BR) | JX064930 | JX064976 | JX065029 |
| Endophyte of *Rytigynia umbellulata* | Lachenaud et al 852 (BR) | JX064926 | JX064973 | JX065025 |
| Endophyte of *Vangueria cinerascens* | Dessein et al 202 (BR) | JX064942 | JX064978 | JX065030 |
| Endophyte of *Vangueria cinerascens* | Dessein et al 443 (BR) | JX064943 | JX064977 | JX065031 |
| Endophyte of *Vangueria dryadum* | Lemaire & Verstraete 289 (BR) | JX064939 | JX064980 | JX065033 |
| Endophyte of *Vangueria infausta* | Dessein et al 879A (BR) | JX064959 | JX064988 | JX065041 |
| Endophyte of *Vangueria infausta* | Dessein et al 879B (BR) | JX064960 | JX064989 | JX065042 |
| Endophyte of *Vangueria infausta* | Dessein et al 879C (BR) | JX064961 | JX064990 | JX065043 |
| Endophyte of *Vangueria infausta* | Dessein et al 879D (BR) | JX064962 | JX064991 | JX065044 |
| Endophyte of *Vangueria infausta* | Lemaire & Verstraete 13 (BR) | JX064951 | JX064992 | JX065045 |
| Endophyte of *Vangueria infausta* | Lemaire & Verstraete 21 (BR) | JX064954 | JX064993 | JX065047 |
| Endophyte of *Vangueria infausta* | Lemaire & Verstraete 31 (BR) | JX064955 | JX064994 | JX065049 |
| Endophyte of *Vangueria infausta* | Lemaire & Verstraete 38 (BR) | JX064957 | JX064995 | JX065050 |
| Endophyte of *Vangueria infausta* | Lemaire & Verstraete 51 (BR) | JX064952 | JX064999 | JX065051 |
| Endophyte of *Vangueria infausta* | Lemaire & Verstraete 61 (BR) | JX064958 | JX064996 | JX065052 |
| Endophyte of *Vangueria infausta* | Lemaire & Verstraete 207 (BR) | JX064953 | JX064997 | JX065046 |
| Endophyte of *Vangueria infausta* | Lemaire & Verstraete 279 (BR) | JX064956 | JX064998 | JX065048 |
| Endophyte of *Vangueria lasiantha* | Lemaire & Verstraete 209 (BR) | JX064940 | JX064981 | JX065034 |
| Endophyte of *Vangueria lasiantha* | Lemaire & Verstraete 269 (BR) | JX064941 | JX064982 | JX065035 |
| Endophyte of *Vangueria latifolia* | Lemaire & Verstraete 69 (BR) | JF265205 | JF265182 | JF265228 |
| Endophyte of *Vangueria latifolia* | Lemaire & Verstraete 74 (BR) | JF265206 | JF265183 | JF265229 |
| Endophyte of *Vangueria latifolia* | Lemaire & Verstraete 141 (BR) | JF265204 | JF265181 | JF265227 |
| Endophyte of *Vangueria macrocalyx* | Lemaire & Verstraete 68 (BR) | JX064949 | JX064984 | JX065037 |
| Endophyte of *Vangueria macrocalyx* | Lemaire & Verstraete 71 (BR) | JX064950 | JX064985 | JX065038 |
| Endophyte of *Vangueria macrocalyx* | Lemaire & Verstraete 114 (BR) | JX064948 | JX064983 | JX065036 |
| Endophyte of *Vangueria madagascariensis* | Lemaire & Verstraete 189 (BR) | JX064963 | JX065000 | JX065054 |
| Endophyte of *Vangueria micropyren* | Dessein et al 253 (BR) | JX064944 | JX065016 | JX065057 |
| Endophyte of *Vangueria micropyren* | Dessein et al 929 (BR) | JX064945 | JX065017 | JX065058 |
| Endophyte of *Vangueria parvifolia* | Lemaire & Verstraete 265 (BR) | JX064946 | JX064986 | JX065039 |
| Endophyte of *Vangueria pygmaea* | Dessein et al 666 (BR) | JF265211 | JF265188 | JF265234 |
| Endophyte of *Vangueria pygmaea* | Dessein et al 726 (BR) | JF265212 | JF265189 | JF265235 |
| Endophyte of *Vangueria pygmaea* | Lemaire & Verstraete 26A (BR) | JF265207 | JF265184 | JF265230 |
| Endophyte of *Vangueria pygmaea* | Lemaire & Verstraete 26B (BR) | JF265208 | JF265185 | JF265231 |
| Endophyte of *Vangueria pygmaea* | Lemaire & Verstraete 28 (BR) | JF265209 | JF265186 | JF265232 |
| Endophyte of *Vangueria pygmaea* | Lemaire & Verstraete 42 (BR) | JF265210 | JF265187 | JF265233 |
| Endophyte of *Vangueria randii* ssp *chartacea* | Lemaire & Verstraete 98 (BR) | JX064964 | JX065001 | JX065053 |
| Endophyte of *Vangueria soutpansbergensis* | Lemaire & Verstraete 285 (BR) | JX064947 | JX064987 | JX065040 |
| Endophyte of *Vangueria thamnus* | Bester 10538 (PRE) | JF265213 | JF265190 | JF265236 |
| Endophyte of *Vangueria thamnus* | Lemaire & Verstraete 25A (BR) | JF265214 | JF265191 | JF265237 |
| Endophyte of *Vangueria thamnus* | Lemaire & Verstraete 25B (BR) | JF265215 | JF265192 | JF265238 |
| Endophyte of *Vangueria thamnus* | Steyn 1835 (PRE) | JF265216 | JF265193 | JF265239 |
| Endophyte of *Vangueria triflora* | Lemaire & Verstraete 53 (BR) | JX064938 | JX064979 | JX065032 |
